# *Fitmunk*: improving protein structures by accurate, automatic modeling of side-chain conformations

**DOI:** 10.1107/S2059798315024730

**Published:** 2016-01-28

**Authors:** Przemyslaw Jerzy Porebski, Marcin Cymborowski, Marta Pasenkiewicz-Gierula, Wladek Minor

**Affiliations:** a Department of Molecular Physiology and Biological Physics, University of Virginia, Jordan Hall, 1340 Jefferson Park Avenue, Charlottesville, VA 22908, USA; b Faculty of Biochemistry, Biophysics and Biotechnology, Jagiellonian University, ul. Gronostajowa 7, 30-387 Kraków, Poland

**Keywords:** side-chain fitting, validation, structure building, refinement, automation

## Abstract

Improvements in crystallographic hardware and software have allowed automated structure-solution pipelines to approach a near-‘one-click’ experience for the initial determination of macromolecular structures. However, in many cases the resulting initial model requires a laborious, iterative process of refinement and validation. A new method has been developed for the automatic modeling of side-chain conformations that takes advantage of rotamer-prediction methods in a crystallographic context. The algorithm, which is based on deterministic dead-end elimination (DEE) theory, uses new dense conformer libraries and a hybrid energy function derived from experimental data and prior information about rotamer frequencies to find the optimal conformation of each side chain. In contrast to existing methods, which incorporate the electron-density term into protein-modeling frameworks, the proposed algorithm is designed to take advantage of the highly discriminatory nature of electron-density maps. This method has been implemented in the program *Fitmunk*, which uses extensive conformational sampling. This improves the accuracy of the modeling and makes it a versatile tool for crystallographic model building, refinement and validation. *Fitmunk* was extensively tested on over 115 new structures, as well as a subset of 1100 structures from the PDB. It is demonstrated that the ability of *Fitmunk* to model more than 95% of side chains accurately is beneficial for improving the quality of crystallographic protein models, especially at medium and low resolutions. *Fitmunk* can be used for model validation of existing structures and as a tool to assess whether side chains are modeled optimally or could be better fitted into electron density. *Fitmunk* is available as a web service at http://kniahini.med.virginia.edu/fitmunk/server/ or at http://fitmunk.bitbucket.org/.

## Abstract 2

A new method for automatic modeling of side-chain conformations that takes advantage of rotamer-prediction methods in a crystallographic context can improve the accuracy and validity of crystallographic models.

## 1. Introduction   

Macromolecular crystallography (MX) is a major tool for determining the atomic structures of proteins and protein complexes (Berman *et al.*, 2013 ▸). The structures determined by MX are a central part of protein function studies (Richardson & Richardson, 2014 ▸). The current methodology is very robust and allows the determination of structures of macromolecules of all sizes: from small structures of enzymes (Blake *et al.*, 1965 ▸; Matthews *et al.*, 1967 ▸) to huge assemblies such as virus capsids (Harrison *et al.*, 1978 ▸; Abad-Zapatero *et al.*, 1980 ▸), ribosomes (Ban *et al.*, 2000 ▸; Schluenzen *et al.*, 2000 ▸; Wimberly *et al.*, 2000 ▸) and vault particles (Tanaka *et al.*, 2009 ▸). Information that can be derived from crystal structures also ranges from single protein–small molecule interactions (Wall *et al.*, 1997 ▸; Porebski *et al.*, 2012 ▸), through the determinants of protein complex formation (Buckle *et al.*, 1994 ▸; Niedzial­kowska *et al.*, 2012 ▸), to the mechanistic behavior of molecular factories (Abrahams *et al.*, 1994 ▸). The widespread application of structural data determined by MX in many different fields has made the technique instrumental to many discoveries.

To facilitate the widespread use of MX, in some cases by scientists who are not experts in structural biology, an enormous effort has been put into the development of easy-to-use, nearly automated software that aids researchers during the process of data reduction (Otwinowski *et al.*, 2003 ▸; Kabsch, 2010 ▸) and crystal structure determination and refinement (Panjikar *et al.*, 2005 ▸; Minor *et al.*, 2006 ▸; Sheldrick, 2008 ▸; Winn *et al.*, 2011 ▸; Adams *et al.*, 2013 ▸). Progress in software has made MX accessible to many noncrystallographers by providing automated and semi-automated pipelines that sometimes approach a ‘one-click’ experience for structure determination and initial model building (Wlodawer *et al.*, 2013 ▸). This has allowed MX to play an increasingly pivotal role in various biomedical and biological research areas (Berman *et al.*, 2013 ▸; Giegé, 2013 ▸; Richardson & Richardson, 2014 ▸), and results in a constantly growing flux of new structures deposited into the Protein Data Bank (PDB; Berman *et al.*, 2000 ▸).

The accelerating expansion of the PDB has led to the development of various new tools for structural bioinformatics and data mining, which has resulted in new methods for structure validation that have recently been standardized and applied to the whole repository (Read *et al.*, 2011 ▸). Regardless, there are many structures, both old and newly deposited, of suboptimal quality in terms of model correctness and model-to-data correspondence (Cooper *et al.*, 2011 ▸; Pozharski *et al.*, 2013 ▸; Shabalin *et al.*, 2015 ▸). There are several possible approaches to reduce the number of problems with protein model quality. Examples of these approaches include automated validation, in which users are alerted about problems during refinement (Urzhumtseva *et al.*, 2009 ▸; Cymborowski *et al.*, 2010 ▸), improvement of software in an attempt to eliminate problems prior to human involvement (Headd *et al.*, 2009 ▸; Bell *et al.*, 2012 ▸), or re-refinement using automated, state-of-the-art tools, improving even legacy structures (Joosten *et al.*, 2012 ▸), although automatic re-refinement still has its limitations (Chruszcz *et al.*, 2010 ▸).

Here, we present *Fitmunk*, a new program for the automatic building of amino-acid side chains in protein crystal structures. *Fitmunk* can be used at various stages: model building, model refinement, and the validation and re-refinement of protein models. We expect that wide use of *Fitmunk* will significantly improve the quality of crystallographic models and thus the reproducibility of electron-density map interpretation.

The problem of building a crystallographic model is comparable to theoretical protein modeling, and many of the algorithms used in this field have been adapted to crystallography. A current and very successful example of such an adaptation is *Rosetta*. The development of molecular-replacement protocols using *Rosetta* utilizing electron density to rebuild models has been instrumental in solving otherwise unsolvable structures (DiMaio *et al.*, 2011 ▸). Because the problem of theor­etical protein modeling can be divided into several parts, *e.g.* protein backbone modeling and side-chain packing, algorithms and programs have been developed exclusively for these specific tasks. An example of a specialized program for side-chain conformation modeling that can be used in protein crystallography is *MUMBO* (Stiebritz & Muller, 2006 ▸), which uses a set of deterministic algorithms based on dead-end elimination (DEE) theory (Goldstein, 1994 ▸) combined with stochastic sampling to solve the combinatorial problem.

Both generalized (such as *Rosetta* or *RAPPER*; Furnham *et al.*, 2006 ▸) and specialized (such as *MUMBO*) protein structure-prediction programs use the same conceptual framework for theoretical modeling and crystallographic model building; in the latter case, the fit to the electron density is added as an additional term in their energy functions. In both cases, the set of algorithms used to solve the combinatorial problem is the same. Our program *Fitmunk* uses a slightly different approach. While it is based on DEE theory, we have developed new algorithms designed specifically for building crystallographic models that rely on the highly discriminatory nature of electron-density maps. These algorithms allow *Fitmunk* to use extensive conformational sampling, which improves the accuracy of the modeling, and makes the software a versatile tool for crystallographic model building, refinement and validation.

## 2. Methods   

**Figure 1:**
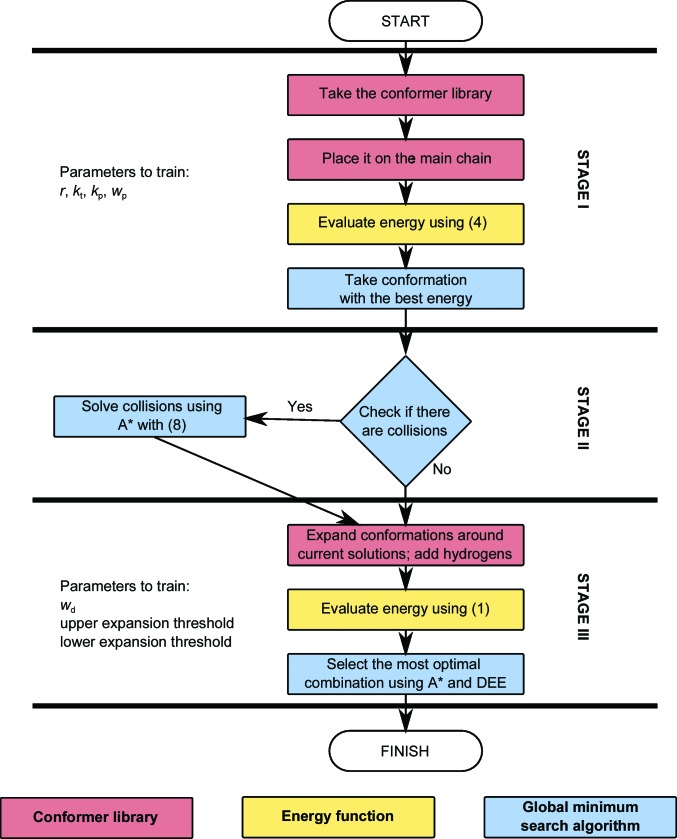
Overview of *Fitmunk* workflow. The overview shows the major steps of the algorithm used by *Fitmunk*. The workflow is divided into three stages: stage I, fitting; stage II, collision resolution; stage III, refitting with pairwise interactions. The parameters that were optimized to achieve the best accuracy are shown on the left. The color of the step corresponds to the component of the algorithm that is responsible for the step.

Electron-density maps derived from experimental X-ray diffraction data can provide very descriptive information about the position of optimal amino-acid side-chain conformations. Unfortunately, for several reasons, including the inadequate quality of many crystals, a lack of high-resolution data and/or incomplete phases, protein electron-density maps are often noisy. To address this issue, the developed algorithm leverages information obtained from electron-density maps and supplements it with prior information about the distribution of amino-acid side-chain conformers. The algorithm used by *Fitmunk* comprises three components: an energy function, conformer libraries and a DEE-based search and collision-resolution algorithm (Fig. 1 ▸), which are described in detail in the following sections.

### 2.1. Energy function   

In *Fitmunk*, the total energy of the system is expressed as
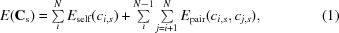
where **C**_s_ specifies a tuple of single conformations for each of the *N* residues of the polypeptide and *c*_*i*,*s*_ is the *s*th conformation of the *i*th residue. *E*_self_ is the self-energy of each conformation, which is defined as
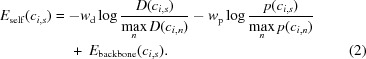
The first term is the energy of conformation *s* fitted to electron density *D*(*c*_*i*,*s*_) relative to the best-fitting conformation of the same residue [max_*n*_ *D*(*c*_*i*,*n*_)]. The second term is the energy of the frequency of conformation *s*, *p*(*c*_*i*,*s*_), relative to the most frequent conformation of the residue from the library [max_*n*_ *p*(*c*_*i*,*n*_)] and the third term is the energy of interaction of the residue in the given conformation with the protein backbone. Similar to the approach of Krivov and coworkers, weighting coefficients *w*_d_ and *w*_p_ are used for the density-fit and frequency terms, respectively (Krivov *et al.*, 2009 ▸). These weighting terms are allowed to be dependent on resolution (for *w*_d_) or resolution and residue type (for *w*_p_).

The following function is used in *Fitmunk* to score the fit of conformation *s* to electron density:
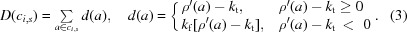
ρ′(*a*) is the value of the electron density for atom *a* sampled at point *a* and at six points around it positioned along each of the axes at distance *r*, averaged and normalized *versus* the r.m.s.d. of the electron-density values. The purpose of introducing a penalty threshold (*k*_t_) and a penalty factor (*k*_f_) is to preferentially score conformations that lie in continuous electron density with a level higher than *k*_t_. *k*_t_, *k*_f_ and *r* are allowed to be dependent on the resolution of the diffraction data. Additionally, penalties are residue type-dependent. Other forms of this function have not been extensively tested; however, the form in (3) gave satisfactory results after the training of penalty parameters. Furthermore, any limitations of this function to reduce the number of potential collisions will be compensated by the collision-resolution step.

The frequency of conformation *s*, *p*(*c*_*i*,*s*_), is calculated on the basis of the backbone-independent (son of) Penultimate Rotamer Library (Lovell *et al.*, 2003 ▸), which provides a probability distribution of side-chain conformations filtered similarly to the Penultimate Rotamer Library (Lovell *et al.*, 2000 ▸). This library is used to assign a frequency of occurrence to each conformation present in the conformer library used by *Fitmunk*. The frequencies of conformations from *Fitmunk*’s library are calculated by interpolating values from frequency sampling provided by Lovell’s library using an inverse-distance weighting method. The obtained values are normalized, so the sum of frequencies for all conformations of a given side chain equals one.

The final term of the self-energy function is the interaction energy between a side chain in a given conformation and the backbone [*E*_backbone_(*c*_*i*,*s*_)]. Nonbonded interactions are represented through Coulomb and Lennard–Jones terms using parameters from the OPLS united-atom force field (Jorgensen & Tirado-Rives, 1988 ▸). To calculate interactions for each side-chain conformation, additional conformations with polar H atoms are generated. H atoms are added using the same geometric criteria as used by *REDUCE* (Word *et al.*, 1999 ▸). For rotatable H atoms, *X*—*X*—O—H torsion angles are set to −60, 60 and 180° for Ser and Thr and to 0 and 180° for Tyr. All three protonation states for His are used.

The second term in the total energy function in (1) [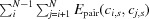] describes the pairwise interaction energy between single conformations of residues, for which only the nonbonded term is used, as described above.

### 2.2. Conformer libraries   

To generate dense conformer libraries, all depositions from the PDB that (i) contained polypeptides longer than ten amino acids, (ii) were deposited together with structure factors between 10 April 2007 and 10 April 2013 and (iii) have a resolution better than 1.8 Å were selected. *R* and *R*_free_ factors were recalculated and 2*mF*_o_ − *DF*_c_ and *mF*_o_ − *DF*_c_ maps were generated using *REFMAC*5 (v.5.7.0029) without any cycles of refinement (Murshudov *et al.*, 2011 ▸). To ensure that the maps to be used for further analysis were recalculated correctly, only those depositions where the recalculated *R* and *R*_free_ factors were within 10% difference from the values reported in the PDB were accepted, with one exception as listed below. For example, a deposition with a recalculated *R*_free_ of 0.22 was accepted if the reported *R*_free_ was 0.20. Depositions that had recalculated *R* and *R*_free_ factors that were both lower than the reported values by more than 10% were also accepted if the difference between the recalculated *R*_free_ and *R* factors was lower than 0.05. Redundancy was removed from the set of accepted depositions by selecting the structure of the highest resolution from each cluster of structures with pairwise identity greater than 40% as clustered by *BLAST* (Altschul *et al.*, 1990 ▸) and provided by the PDB.

Only one chain of a given polypeptide sequence was taken from each deposition. When multiple conformations of a single residue were present, the one with the higher occupancy, or the lower atomic displacement parameters if the occupancies were equal, was selected. 2241 nonredundant chains comprising 438 109 residues were taken for further analysis.

To improve the quality of the conformer libraries, the following side-chain conformations were excluded: those that formed significant ‘clashes’ (*i.e.* van der Waals radii overlaps of greater than 0.4 Å), those that were modeled without significant experimental data (2*mF*_o_ − *DF*_c_ map value for side chain or main chain with *EDSTATS* *Z*-score lower than 1) or those that model the experimental data poorly (significant peaks in the *mF*_o_ − *DF*_c_ map with *Z*-score >3 or <−3). Clashes and map significance values were calculated using *MolProbity* v.3.14.080821 (Chen *et al.*, 2010 ▸) and *EDSTATS* v.0.5 (Tickle, 2012 ▸), respectively. Of 438 109 collected residues, 41 472 were excluded owing to insignificant or misinterpreted electron density. A further 60 387 residues were excluded owing to clashes and 5608 other residues were incomplete, leaving 330 642 for further analysis. The atomic nomenclature of symmetric residues (Arg, Asp, Glu, Phe and Tyr) was normalized by swapping the names of symmetric atoms if particular torsion angles (χ_1_ for Asp, χ_2_ for Glu, Phe and Tyr and χ_5_ for Arg) were outside the range −90 to 90°. Pseudo-chiral residues (Leu and Val) were normalized by swapping the names of equivalent atoms if their ‘chirality’ was reversed. Thr and Ile residues with incorrect chirality were excluded from analysis. For the purpose of clustering, Asn, Gln and His residues were treated as symmetric by applying the same rules as used for symmetric residues and swapping the identities of the atoms of the amide group or imidazole ring. H atoms were removed if present. To remove redundancy at the conformational level, depositions were processed starting from the highest resolution structures, and a new conformation was added to the data set if the r.m.s.d. between the analyzed conformation and previously collected conformations was higher than 0.1 Å (calculated after superimposing the main-chain N, C^α^ and C atoms and measuring deviations over all side-chain atoms excluding C^β^).

Even after filtering out conformations with steric clashes or that have poor fit to electron density, conformations that significantly violated known chemical rules were observed. Therefore, these conformations, specifically those that violated the Engh and Huber target values (Engh & Huber, 2001 ▸) by more than 6σ, were filtered out. Conformers where the position of C^β^ differed by more than 5σ from the mean were also excluded. Finally, we visually inspected conformers that were observed only once and removed serious outliers.

We call this nonredundant filtered data set cl0.1. It contains 83 042 conformations and is a very dense conformer library, which in most cases is too complex for direct use in side-chain conformation modeling. This data set was further clustered using hierarchical agglomerative clustering using the r.m.s.d. of all side-chain atoms (excluding C^β^) as a distance metric, and the maximum linkage criterion as implemented in the *hcluster* Python package (Eads, 2008 ▸). A medoid (the conformation with the smallest sum of r.m.s.d. values between itself and all other conformations in a cluster, weighted by the number of occurrences of each conformation) was selected as a representative conformation for a given cluster. A library that is referred to as cl*X* contains representative conformations, each of which represents a cluster of conformations with a maximum pairwise r.m.s.d. lower than or equal to *X* Å.

Based on the hierarchical clustering process, it is possible to create conformer libraries with lower complexity (*e.g.* cl1.0), which can be dynamically expanded to denser ones (*e.g.* cl0.5). The positions of atoms from all conformations and linkage data are stored in a file in HDF5 format (The HDF Group; http://www.hdfgroup.org/HDF5/), which is designed for fast retrieval.

### 2.3. Dead-end elimination algorithm, collision detection and resolution   

To utilize the complex conformer libraries, it was necessary to design a three-step algorithm (Fig. 1 ▸) with decreasing conformational complexity at each step of the search process. A simplified variant of the self-energy function in (2), where the nonbonded energy term *E*_backbone_(*c*_*i*,*s*_) is removed, is used in the first and second stages to perform faster calculations. In the third stage, however, after reducing the overall complexity in the first two steps, the full energy function in (2) is used as described above. During the first two stages of the search, conformations which form close contacts (clashes) with either the protein backbone (first stage) or other modeled conformations (second stage) are removed. Incorporation of a check for steric clashes was necessary because the simplified self-energy function does not contain any repulsive terms that penalize close contacts. It is possible to represent the energy function with clash elimination as
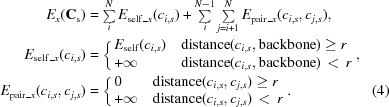



A *k*-d tree is used to search for main-chain or C^β^ atoms that are nearest to the given conformation. The distance cutoff *r* at which distance a conformation is marked as clashing with the backbone depends on the identities of the pair of evaluated atoms. These cutoff distances are calculated as the first percentiles of distances observed in the protein structures that were used for extracting conformations and may be adjusted by a user-selectable distance. By default, these distances are reduced by 0.3 Å to compensate for main-chain position errors during model building. *Fitmunk* also provides an option to remove conformations that clash with other modeled nonproteinaceous waters, ions or small molecules with their own distance thresholds. Not every conformation is evaluated for clashes, because for purposes of efficiency ‘lazy’ evaluation is used, *i.e.* a conformation is only assessed for clashes when it is selected. If the lowest energy conformation does not form clashes, no further conformations are evaluated. In the first stage, clashes between side chains are not considered; therefore, according to (4), the total energy of the conformation is equal to *E*_self_*x*_. This is a special case of the DEE theory. For example, the Goldstein elimination criterion becomes the simple selection of the conformation with the lowest *E*_self_*x*_, 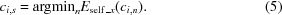



At this point, most of the conformations of the core residues at high and medium resolutions will be properly placed, but several residues will form clashes either inside the asymmetric unit and/or with crystallographically related copies. These collisions are resolved in the second stage.

At the collision-resolution step (the second stage), the simplified energy function in (4) is used as well. Its advantage is that its value does not depend on pairwise interactions, except for colliding conformations. Because colliding conformations are excluded, it is therefore easy to calculate lower energy bounds that can be used as an additional eliminating criterion. For conformations without collisions the elimination criterion becomes
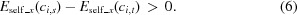



Here, the term is calculated with the remaining residues in ‘compatible’ (*i.e.* nonclashing) conformations and it is always equal to or higher than *E*_low_(·), which is calculated with the remaining residues adopting a conformation with the lowest possible energy, disregarding any clashes. Therefore, it is possible to introduce an elimination criterion based on this lower bound,
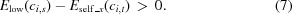
Although this criterion is weaker (permitting more conformations to pass) than (6), the advantage of (7) is that it is possible to eliminate all remaining conformations in a ranked data set if one reaches a conformation that has *E*_low_(*c*_*i*,*s*_) > *E*_self_(*c*_*i*,*t*_), where *c*_*i*,*t*_ is the conformation which gives the lowest energy so far, without actually calculating any collisions.

**Figure 2:**
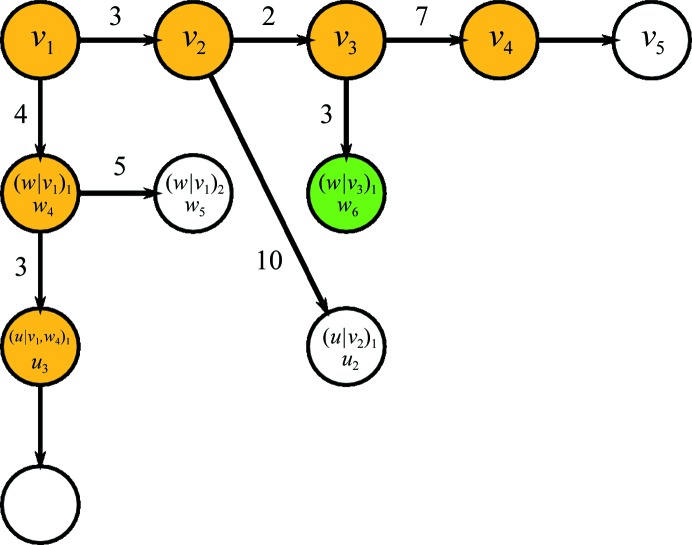
Example of the graph explored by the A* search. The first node *v*_1_ is visited and checked for collisions. It represents the lowest energy conformation of residue *v* but clashes with conformation *w*_1_ and cannot be a terminal node. There are two options: either we choose to keep *v*_1_ and find a nonclashing conformation of *w* (*w*_4_) or we move to the next conformation of *v*. The estimated cost of moving to *v*_2_ is 5, whereas that of remaining at *v*_1_ is 7. *v*_2_ is therefore selected. At this point the following moves are possible: go to *v*_3_ (cost 8), keep *v*_2_ (cost 13) or skip to *v*_1_ and *w*_4_ (cost 7). Because of the lowest cost the last possibility is explored with further options, *w*_5_ (9) and *u*_3_ (10), and previous ones, *v*_3_ (8) and *v*_2_ (13). *v*_3_ is selected and *w*_2_ is evaluated for clashes. Because this does not form any additional collisions it is marked as a terminal node, its estimated cost is used to set *E*_low_ = *E*_self_*x*_, it is possible to eliminate all other conformations based on the criterion in (7) (other evaluated options have costs higher than the final cost of a terminal node) and the selected path leading to the green node is the best solution. During graph exploration, the nodes marked in yellow or green are evaluated for clashes while those in white are not.

To find and resolve collisions, a directed graph is used to describe the relationship between different conformations (nodes *v*, *w*) of one residue [horizontal edges *e*_h_(*v*_*i*_, *v*_*i*+1_)] and the dependency of the conformation of one residue on the conformation of another [vertical edges *e*_v_(*v*_*i*_, *w*|*v_i_*)]. In such a graph, each node has a maximum of two outgoing edges: *e*_v_ and *e*_h_. The A* search algorithm is used to explore this graph (Fig. 2 ▸). Each residue is assigned the lowest energy conformation from the first stage and these conformations are then checked for collisions. To detect collisions between all symmetry-related copies it is necessary to recreate all 27 unit cells. To reduce the complexity of the problem, a bounding sphere around the current conformation of each residue is calculated, the sphere centres are transformed to recreate 27 unit cells and the *k*-d tree algorithm is then used to detect colliding spheres. Finally, pairwise atom distances are checked to determine whether a given pair of conformations actually clash. If no collision is found, according to (4) the residue remains in the lowest energy conformation. If a collision with another residue *w* is found, then the conformation *w*|*v_i_*, which is a conformation of *w* compatible with *v_i_*, is added to the graph and connected with the vertical edge (*v*_i_, *w*|*v_i_*).

A* searches require a heuristic function to estimate the minimal cost *h* of moving to the next node. We define this function as
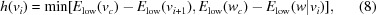
where *v_c_* is the current best conformation. It means that the minimal cost is either an estimated increase in energy after adopting the next ranked conformation of the same residue or an estimated increase after selecting the evaluated conformation together with a noncolliding conformation of residue *w*. If conformation *v_i_* does not clash with any residue, it is marked as a terminal node and selected as a possible candidate for solution.

When a node is visited, the actual cost of moving to this node is calculated as *g*(*v_i_*) = *g*(parent) + *E*_low_(*v*_*c*_) − *E*_low_(*v_i_*) and the estimated total cost of visiting the following nodes as *f*(*v_i_*) = *g*(*v_i_*) + *h*(*v_i_*).

The search is finished when all remaining discovered nodes have an estimated cost higher than the cost of moving to the node already marked as terminal. The nodes forming the path to the terminal node determine the conformations of the respective residues that form a globally optimal solution according to the criterion in (6).

After the second stage of fitting, all of the side chains adopt conformations that minimize the simplified energy function, and in general fit well to the electron-density map. At this point most of the side chains may be brought to the correct positions by crystallographic refinement. The energy function used up to this point did not include nonbonding interactions between residues; therefore, the hydrogen-bond network needs to be optimized by applying flips (if needed) to the side chains of Asn, Gln and His residues.

In the third stage of fitting, the full energy function in (1) is applied, comprising electron-density fit, rotamer frequency and nonbonding interaction terms. The conformations that were determined in the two previous stages are placed near the optimal positions; however, the precision depends on the density of the conformer library sampled in the search process. During the third stage, additional samples from a denser and higher complexity variant of the conformer library centred on the previously established conformation are used to improve conformational sampling. Cluster K1 is defined as that from which the optimal conformation *c*_*i*,*s*_ was taken. To expand the conformations around *c*_*i*,*s*_, cluster K2, which is a superset of K1 at lower complexity (*e.g.* cl1.5), is selected. The hierarchy tree is then traversed down and descendants of K2 at higher complexity (*e.g.* cl0.4) are selected. The conformation sets of Asn, Gln and His residues are also expanded by applying flips if needed, and conformations of residues that contain polar H atoms are further expanded by adding H atoms as described previously.

The self-energies and pairwise interaction energies are calculated for all conformations and a DEE cycle is then performed by sequentially applying multiple criteria: Goldstein (Goldstein, 1994 ▸), single-conformation split DEE (Pierce *et al.*, 2000 ▸) and two-conformation ‘magic bullet’ split DEE (Gordon & Mayo, 1998 ▸; Pierce *et al.*, 2000 ▸). Each DEE criterion is applied iteratively until no further conformations are eliminated before continuing on with the next criterion. The elimination stops after ‘magic bullet’ DEE converges. Residues for which DEE did not identify a unique conformation are then clustered based on the presence of pairwise interactions. Clusters that are smaller than 15 residues are resolved using another A* search. To resolve larger clusters, an additional cycle of DEE is applied. This cycle starts with a DEE based on conformation pairs (Lasters *et al.*, 1995 ▸) and uses the Goldstein criterion. Finally, the cycle for singles is repeated and the remaining interactions are resolved by an A* search. The size of the cluster for which the A* search is applied was determined empirically, *i.e.* our current implementation of A* is faster than DEE for clusters of this size.

### 2.4. Preparation of training and test sets for side-chain modeling   

Two sets of models of protein structures were generated: the first as a training set to optimize tunable energy-function parameters and the second as a test set to estimate the accuracy of *Fitmunk*. The primary data set from which these sets were drawn includes models of structures for which it was possible to recalculate *R* factors close to the reported values (as described in §[Sec sec2.2]2.2), that share no more than 40% identity, that were deposited between 2007 and 2013 and that have a reported resolution between 0.9 and 3.5 Å. From this primary set, 150 structures per resolution bin were randomly selected. From all models, all ligand, ion and water atoms were removed and the models were then subjected to five cycles of *REFMAC* refinement with local automatic NCS and without TLS. Models were divided randomly within each bin, assigning 50 structures to a training set (TR) and 100 structures to a test set (TS). The width of resolution bins was 0.2 Å, except for the highest and lowest resolution bins, for which the ranges 0.9–1.2 and 3.0–3.5 Å were used, respectively. For testing purposes, we also created derivative test sets based on each TS by introducing noise and reducing the completeness of the model. Noise was introduced by changing the χ angles of randomly selected residues by a random rotation between −40 and 40°. Model completeness was reduced by removing the side chains of randomly selected residues. The percentages of side chains that were deleted (*X*) or randomized (*Y*) are indicated by the name of the test set: TS_d*X*_r*Y*. These models were subjected to five cycles of *REFMAC* refinement and the resulting 2*mF*_o_ − *DF*_c_ electron-density maps were used for *Fitmunk* evaluation.

In addition to the test sets based on PDB depositions described above, *Fitmunk* was also used in the determination of 115 new structures solved in the authors’ laboratory. These structures may be considered to be a measure of the real-world performance of the methodology. *Fitmunk* was used at various stages of model building, and included structures determined both by molecular replacement and by experimental phasing. For consistency in comparison, each of the 115 models was refined using *REFMAC* for five cycles without TLS and with automatic local NCS. The resulting models were subjected to *Fitmunk* refinement. Both the reference models and the models after *Fitmunk* side-chain fitting were subsequently refined using *REFMAC* for five cycles. The refined models were then compared in terms of *R*, *R*_free_, *MolProbity* clashscore and *MolProbity* score.

### 2.5. Accuracy metrics and optimization of fitting parameters   

The algorithm described above introduces several tunable parameters that have to be optimized to achieve the best side-chain fitting accuracy. As a measure of accuracy, we selected the percentage of the side chains of a given residue type where the fitted conformation deviated from the original position by an r.m.s.d. of no greater than 0.5 Å. If the optimized parameter is independent of residue type, the accuracy calculated for all residues together was used as a metric. For training purposes Asn, Gln and His residues were treated as symmetric, except in the nonbonded interaction weight-optimization step. For the purpose of checking the correct Asn, Gln and His conformations, the orientations of these residues in the reference structures were optimized using *REDUCE* from the *MolProbity* suite.

To determine values of the energy-function parameters that maximize the accuracy, a grid test was performed on the TR sets. The obtained values were smoothed assuming continuous resolution dependency using multiquadratic radial basis functions (RBFs), with ∊ set to 2. The values that maximized the accuracy were chosen and then tested using the TS sets. If the improvement was insignificant (below 1% of improved conformations for a given residue type in the resolution bin), neutral values were chosen (0 for *k*_t_, 1 for *k*_f_, 0 for *r* and 10^−5^ for *w*_d_/*w*_p_). The search for optimal parameter values was split into several independent steps associated with each energy term. Firstly, the values of the parameters of the electron-density-related term were optimized. These are the averaging radius (*r*), the penalty threshold (*k*_t_) and the penalty factor (*k*_f_). Optimization was performed using only the electron-density-related term from the energy function, using the cl0.7 conformer library. The cl0.7 conformer library was selected because it offered a reasonable accuracy to speed ratio. To independently evaluate all amino acids, optimization was performed without collision resolution (only the first stage of fitting was performed). The averaging radius value was optimized independently of the *k*_t_ and *k*_f_ values, which were optimized simultaneously thereafter, followed by optimization of the *w*_d_/*w*_p_ ratio. Because RBF smoothing of the trained weights ratio produced sub­optimal results, the values that maximized the accuracy on the training set were used directly instead of the values obtained through smoothing. All of these parameter values were dependent on resolution and residue type.

As the nonbonded interaction terms were not yet used at this point, it was sufficient to optimize the ratio of the *w*_d_ and *w*_p_ weights. Their values relative to the final energy term were established in the final optimization step in a residue-type-independent manner. The training of the absolute value of these weights was performed using different upper and lower thresholds for expanding the search space.

Because the training of the final values of the weights and the optimization of the cluster-expansion thresholds did not improve the accuracy significantly, cluster expansion was not used for testing the accuracy of the determination of the proper Asn, Gln and His orientations. Instead, only the addition of polar H atoms and the generation of both orientations of Asn, Gln and His were performed.

## 3. Results and discussion   

*Fitmunk* was primarily designed to be used at different stages of protein model building, structure refinement and validation. The *Fitmunk* program has also been used for the identification of peptide sequence from even relatively poor electron-density maps (Niedzialkowska *et al.*, 2015 ▸). Here, we focus on the application of *Fitmunk* to protein model building and validation and show the impact of *Fitmunk* on the quality of the resulting models, the completeness of the models for subsequent computational studies and, in some cases, re-evaluation of the biological roles of some amino-acid residues.

### 3.1. Model building   

*Fitmunk* was initially developed to build amino-acid side chains during automated building of macromolecular models to electron density, and the configurable parameters of the program were optimized for structures that are in the late stages of refinement. However, during program development our group started to routinely use *Fitmunk* even during the initial stages of model building and refinement. To date, the program has been tested on 65 different proteins (115 total different data sets), improving the model refinement of serum albumins (Majorek *et al.*, 2012 ▸), histone code reader proteins (Niedzialkowska *et al.*, 2012 ▸), complexes of antibodies with allergens (Chruszcz *et al.*, 2012 ▸) and several enzymes (Porebski *et al.*, 2012 ▸; Shabalin *et al.*, 2012 ▸). *Fitmunk* has also been used for the structure determination of many other protein structures from several structural genomics centers, including CSGID, MCSG and NYSGRC, as well as for the re-refinement of previously determined structures in the PDB. However, the performance of the program was not systematically evaluated in all of these cases.

**Figure 3:**
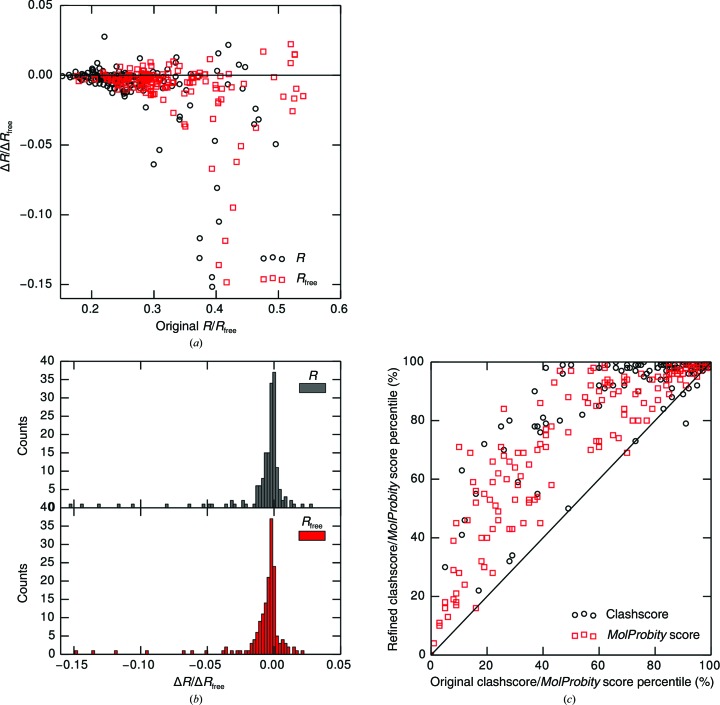
Changes in global parameters after refinement of structures with *Fitmunk*. 115 different structures of 65 different proteins are represented. (*a*) Changes in *R* (black circles) and *R*_free_ (red squares) after *Fitmunk* refinement. (*b*) Histograms of Δ*R* (gray) and Δ*R*_free_ (red) between the original and the *Fitmunk*-refined structure. (*c*) Changes in *MolProbity* clashscore percentiles (black circles) and *MolProbity* score percentiles (red squares) shown as a value of the parameter calculated for the *Fitmunk*-refined structure *versus* the value of the parameter calculated for the unrefined structure. All parameters are calculated using the original structure after five cycles of *REFMAC* refinement and the original structure refined with *Fitmunk* and five cycles of *REFMAC*. Black lines in (*a*) (horizontal line) and (*c*) (diagonal line) represent no change in a parameter.

**Figure 4:**
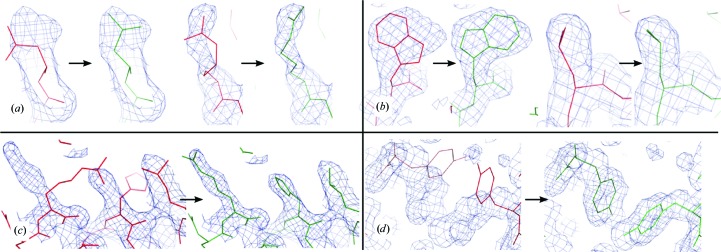
Examples of side-chain position improvement after rebuilding by *Fitmunk*. (*a*) Improving the fit by selecting a different side-chain conformation. (*b*) Similar fit using a better rotamer. (*c*) Fixing cascade errors. (*d*) Fixing conformations of an MR model where the previous model had different crystal contacts. In all panels, the red lines represent the model before and the green lines represent the model after rebuilding by *Fitmunk*. Electron-density maps are 2*mF*_o_ − *DF*_c_ maps contoured at the 1 r.m.s.d. level

Fig. 3 ▸ shows how *Fitmunk* improved the model-to-data correspondence, the model quality and the model completeness as evaluated using the 115 selected structures cited above. Manual inspection of the models showed that *Fitmunk* significantly improved different aspects of these models (Fig. 4 ▸): it was able to select better conformations and better rotamers with a similar fit, to resolve cascade errors and to introduce conformational changes related to differences in crystal contacts or bound ligands in models used for molecular replacement (MR). We also investigated how refinement with *Fitmunk* affected *R* and *R*_free_, the *MolProbity* percentile clashscore and the overall *MolProbity* percentile score. *R* and *R*_free_ were influenced the most when many side chains were not present in the original model [for example, after MR with a model generated by *CHAINSAW* (Stein, 2008 ▸), which removes all side chains or some side-chain atoms on the basis of sequence alignment between the target sequence and the model used for MR] and were rebuilt by *Fitmunk*. In such cases we observed a drop in *R*_free_ of as high as 0.15 when only the backbone of the protein was used. It was possible to successfully use *Fitmunk* even with relatively incomplete structures that had an initial *R*_free_ of <0.45; however, the best improvement was achieved with structures that had a well modeled backbone and an *R*_free_ of ∼0.4 or better (Fig. 3 ▸). For models that were near the final stages of refinement (*R*_free_ of <0.2 and all side chains already modeled) the improvement in *R* and *R*_free_ averaged 0.003 (16 models). 

The main strengths of *Fitmunk* are observed when analyzing model quality. Even for nearly final structures, for which *Fitmunk* did not affect the *R* factors significantly, application of the side-chain-fitting algorithm considerably improved the geometry by reducing the clashscore and the number of bad rotamers. In the initial data set only 60% of the structures had a clashscore in the 90th percentile and only 20% had a *MolProbity* score in the 90th percentile when compared with structures of similar resolution. After refinement with *Fitmunk* 84% of structures had a clashscore and 46% had a *MolProbity* score in the respective 90th percentiles (Fig. 3 ▸).

To systematically assess the broad applicability of *Fitmunk*, we tested the program on a large (1100) subset of structures deposited in the PDB with a fraction of their amino-acid side-chain conformations randomly changed or removed. These artificial test sets cover a broad range of different stages of model building and refinement, and vary significantly in the type of protein and diffraction data quality. Additionally, by systematically increasing the number of model perturbations and by decreasing the model completeness we were able to simulate how *Fitmunk* will behave in iterative refinement scenarios.

**Figure 5:**
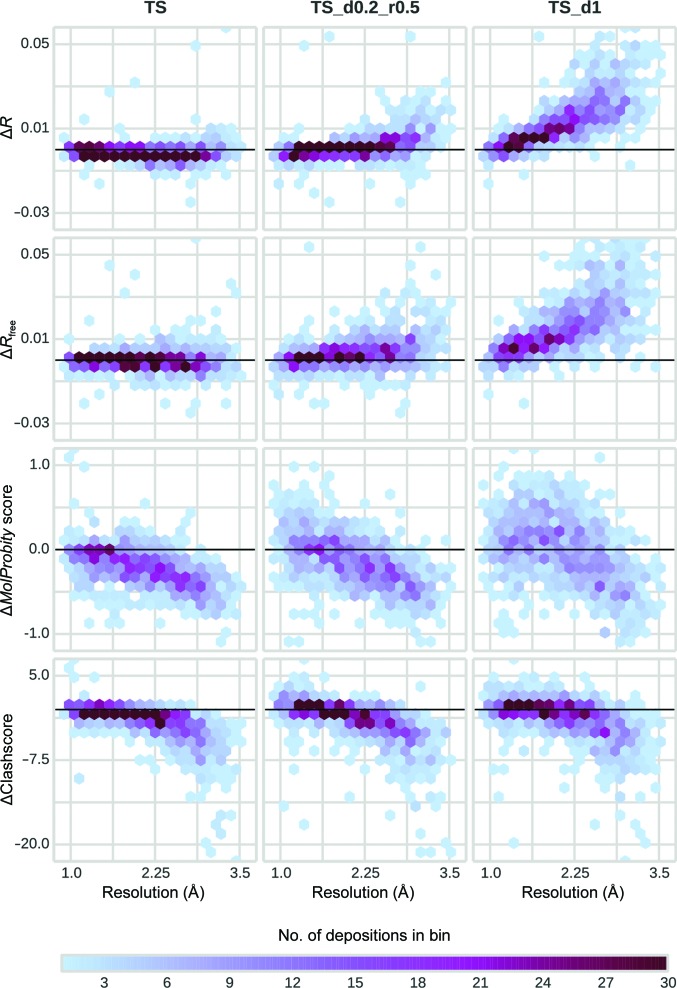
Changes in *R*, *R*_free_, *MolProbity* clashscore and *MolProbity* score *versus* resolution after the refinement of different test sets with *Fitmunk* compared with a reference test set. The values are represented as a two-dimensional histogram using hexagonal bins. The color of each bin encodes the number of models that fall within it. *Fitmunk* was able to reproduce *R* and *R*_free_ and improve the *MolProbity* score and clashscore (lower is better) even when noisy and incomplete test sets were used. Supplementary Table S1 ▸ shows the median changes for each analyzed resolution bin for the TS test sets. Black horizontal lines represent no change in the analyzed parameter.

Firstly, we observed that refinement using *Fitmunk* was able to match or improve the *R*_free_ of almost 50% of structures from the fully refined test set (TS). Most of the remaining structures had an *R*_free_ that was at most 0.01 higher than the unrefined value. For the same set, *Fitmunk* was able to improve the *MolProbity* percentile score (which in the case of *Fitmunk* refinement is mostly improved by the selection of better rotamers and changes in the number of steric clashes); more than 80% of the structures had improved *MolProbity* percentile scores. For the sets that were progressively less complete and accurate, *Fitmunk* was able to reproduce similar or better *R*_free_ values and *MolProbity* scores when compared with the original, refined structures, even when half of the side chains were missing (for this set, 70% of the structures refined to *R*_free_ values within 0.01 or better than the original). The only test set for which most of *Fitmunk*-refined structures (>70%) failed to refine to similar *R*_free_ values to those of the original structures was the test set in which only the backbones of the proteins were present. Removal of all side chains significantly affects the quality of the electron-density map, and therefore such behavior was expected. Even then, 90% of structures have *R*_free_ values that are within 0.02 of the original. Similar behavior was observed for the *MolProbity* scores (Fig. 5 ▸ and Supplementary Table S1).

**Figure 6:**
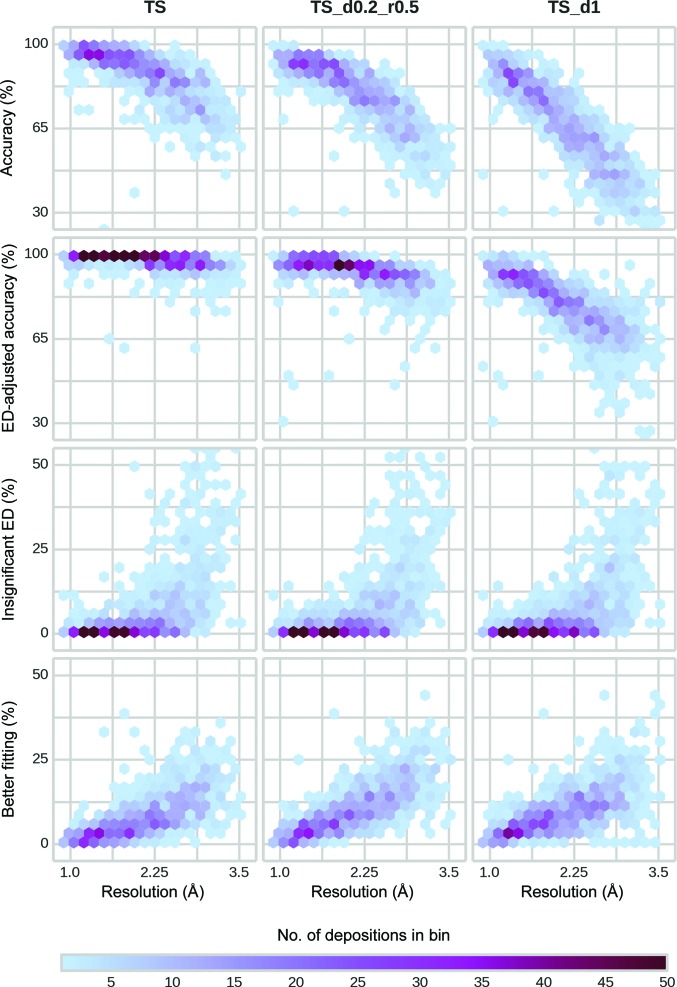
Accuracy of *Fitmunk* side-chain modeling on different test sets. The values are represented as two-dimensional histograms using hexagonal bins. The color of each bin represents the number of models that fall within it. The accuracy (calculated with the assumption that the original depositions are correct) of *Fitmunk* was on average between 96.5 and 70.8% per deposition depending on resolution, while on average the ED-adjusted accuracy (taking into account that for some residues *Fitmunk* was selecting a conformation that fitted better to the electron density or that the electron density was so weak that it did not mandate any conformation) was between 99.5 and 95.7%. *Fitmunk* was also able to achieve high accuracy for highly incomplete structures (TS_d0.2_r0.5 test set) and reasonable accuracy for backbone only (TS_d1). Depending on the resolution, *Fitmunk* was able to improve the fit of 1.7–16.8% of residues compared with the original depositions. Supplementary Table S2 shows the median accuracies, median percentages of residues with insignificant ED and median percentage of residues with improved fit for each analyzed resolution bin for the TS set.

We also assessed the local accuracy of *Fitmunk* refinement (Fig. 6 ▸). The side-chain conformations of the original protein models (as deposited) were compared with the conformations present in the models rebuilt by *Fitmunk*. We compared the structures using two different assumptions: (i) the models present in the PDB are locally optimal and should be treated as the final target or (ii) the models present in the PDB are sometimes locally suboptimal and at some positions a conformation may be found that better explains the electron density (or the electron density may be insignificant due to the side-chain disorder, so any modeled conformation would not be justified). We therefore measured two types of accuracy. Accuracy that assumes (i) above is calculated as a percentage of all residues (excluding Ala and Gly) that have all of their χ angles within 20° of the original conformation. Accuracy that assumes (ii) above, and thus is adjusted by electron density (ED), is calculated as the percentage of residues with significant density (calculated either before or after *Fitmunk* refinement, with a *Z*-score of the density occupied by side chain as reported by *EDSTATS* to be >1 for a 2*mF*_o_ − *DF_c_* ED map) that are either the same as original conformation (within 20°) or are different from the original conformation but are occupying more significant density than the original conformation. The accuracy of *Fitmunk* was on average between 96.5 and 70.8% per deposition depending on resolution, while on average the ED-adjusted accuracy was between 99.5 and 95.7%. If the side chain of a residue had significant ED, *Fitmunk* was able to select a conformation that fitted better to the ED than the deposited conformation for between 1.7 and 16.8% of residues, depending on resolution. For some depositions the number of improved residues was greater than 30% (Fig. 6 ▸ and Supplementary Table S2).

We have also compared the accuracy of *Fitmunk* with the reported accuracy of *MUMBO*. As *MUMBO* is a side-chain-fitting program that utilizes DEE-based algorithms similar to those of *Fitmunk*, but uses a typical protein-design framework with an additional energy term corresponding to the fit to ED, this comparison demonstrates the advantage of using algorithms that were specifically developed for crystallography. The results presented in Table 1 ▸ indicate that *Fitmunk* out­performed *MUMBO* in all tested cases.

There was a significant fraction of structures that were improved by refinement with *Fitmunk* when compared with the originally deposited structure. Almost 50% of the structures from the TS sets have their *R*_free_ improved, and more than 80% of these have better geometry as measured by the *MolProbity* percentile score. This indicates that *Fitmunk* is a useful tool for the re-refinement and validation of older structures.

### 3.2. Model validation   

We have tested *Fitmunk* using the TS set for three types of validation: (i) validation of conformer selection, (ii) validation of missing side chains and (iii) validation of main-chain positioning. By default, *Fitmunk* rebuilds all side chains regardless of the level of disorder. Therefore, after side-chain fitting and subsequent refinement, we assessed the fit to the electron density with *EDSTATS*. Similarly to the criteria for the ED-adjusted accuracy, we treat side-chain conformations as supported by ED when the significance (*Z*-score) of the density occupied by the side chain as reported by *EDSTATS* is >1 for the 2*mF*_o_ − *DF*_c_ ED map and if there is no significant (*Z*-score >3) difference density (either positive or negative). The new side-chain conformation was considered to better correspond to the electron density than that from the original deposition when it occupied significant density and when the *EDSTATS* significances were higher for the 2*mF*_o_ − *DF*_c_ map and lower for the *mF*_o_ − *DF*_c_ map than for the corresponding values for the original model.

**Figure 7:**
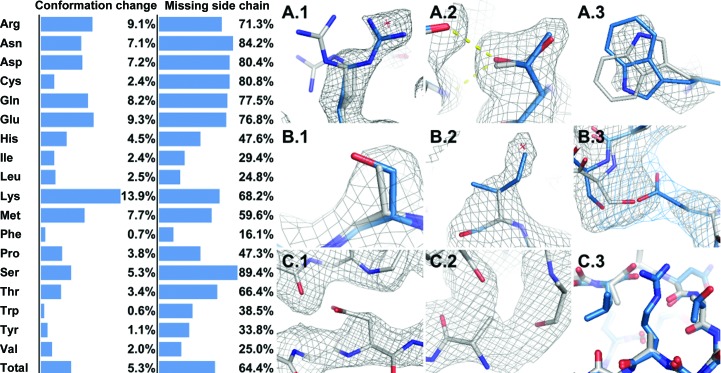
The results of *Fitmunk* refinement can be used for different types of validation. In the left panel, ‘conformation change’ gives the fraction of all side chains (by residue type) that had their conformation changed by *Fitmunk* and had an improved fit to electron density as evaluated by *EDSTATS* after *REFMAC* refinement; ‘missing side chain’ gives the fraction of missing side chains that were rebuilt by *Fitmunk* and were fitted to significant electron density as evaluated by *EDSTATS*. Right panel, examples of corrections introduced into models and problems spotted resulting from *Fitmunk* refinement. Original models are represented as white sticks and *Fitmunk*-corrected models as blue sticks. Original 2*mF*_o_ − *DF*_c_ density at the 1 r.m.s.d. level is displayed as a gray mesh. In selected cases 2*mF*_o_ −*DF*_c_ electron density resulting from the refinement of a *Fitmunk*-corrected model is displayed at the 1 r.m.s.d. level as a blue mesh. Top row, conformational changes introduced by *Fitmunk*. A.1, correction of wrong conformation selection: several alternative conformations and a water were replaced by better fitting conformations. A.2, selection of a rotamer that forms better interactions. A.3, selection of a conformation that has a better density fit. Middle row, missing side chains that were rebuilt by *Fitmunk*. B.1, small residues such as Ser can be rebuilt most of the time. B.2, missing side chains were improperly compensated for by the introduction of water. B.3, rebuilding of missing residues allowed better packing and better main-chain placement after refinement. Bottom row, problems spotted after *Fitmunk* was unable to fit any conformation or resolve steric clashes for a given residue. C.1, possible wrong sequence or register shift; *Fitmunk* was unable to fit Asp in the space available. C.2, main-chain break and wrong main-chain orientation; *Fitmunk* was unable to fit any conformation of Ile with the given backbone orientation. C.3, wrong loop conformation; *Fitmunk* was unable to resolve collisions in the tight loop because of the incorrect backbone conformation.

**Figure 8:**
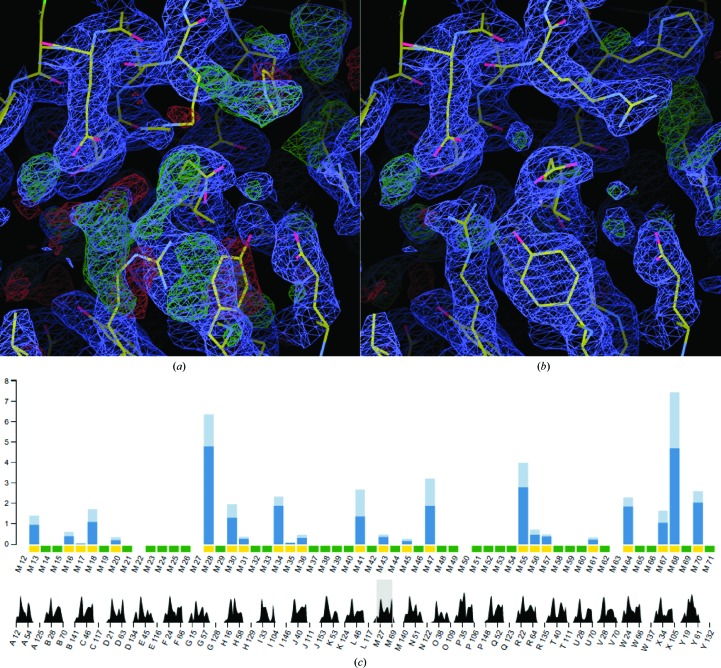
Example of the incorrect interpretation of an electron-density map that can be detected on the basis of multiple side-chain conformation changes made by *Fitmunk*. (*a*) Original deposition with PDB code 4emh (yellow lines) shown with 2*mF*_o_ − *DF*_c_ (blue; at the 1 r.m.s.d. level) and *mF*_o_ − *DF*_c_ (green, positive; red, negative; both at the 3 r.m.s.d. level) electron-density maps. Multiple regions of low model–data correspondence can be seen. (*b*) Model refined with *Fitmunk*. All incorrectly fitted side chains have been corrected. (*c*) Fragment of a screenshot from the *Fitmunk* server showing differences between the original model and the model refined with *Fitmunk*. Analysis of regions with multiple differences led to the identification of the fragments shown in (*a*) and (*b*).

If *Fitmunk* selects a better fitting side-chain conformation than that present in the original deposition it means that the selection of the conformer may be suboptimal. This can be used for validation of conformer selection. We observe that in our test data set about 5% of the residues may have a suboptimal conformation (Fig. 7 ▸). Changes that are introduced to many neighboring residues may indicate more serious problems that can affect interpretation of the model. For example, one of the significant cases involved an RNA-binding protein that forms different oligomeric states in solution (Wu *et al.*, 2012 ▸). The authors of the deposition (PDB entry 4emh) wrongly modeled the conformations of side chains in different parts of the protein that were involved in forming the interfaces (Fig. 8 ▸). Moreover, reinterpretation of the map in the affected regions showed that the N-terminal end of the protein was placed in the wrong direction. Fortunately, the authors did not make any conclusions based on the model itself, but based them on other experimental data. However, when the original structure was analyzed with *PDBePISA* in order to predict the probable oligomeric state, no stable oligomers were identified. When the structure was corrected the dimers were identified and trimers were more energetically favorable, which was more consistent with other experimental data.

If an original deposition does not contain a side chain at a particular position, or the side chain is incomplete, a conformation selected by *Fitmunk* and supported by ED may indicate that the residue was excluded from the model unnecessarily and the side chain could be rebuilt. This feature of *Fitmunk* may be used to validate whether a side chain should be truncated. In the test data set, about 1% of all residues (64% of incomplete residues) could be modeled into electron density meeting *EDSTATS* criteria.

Finally, *Fitmunk* tries to place side-chain conformations on a fixed backbone. We observed that *Fitmunk* is sometimes not able to select a side-chain conformation that does not collide (even when generous distance cutoffs for clash consideration are used) with the backbone or another neighboring side chain if the backbone is incorrect or suboptimal. Such situations can also occur when the sequence is assigned incorrectly, *e.g.* if Lys is assigned in the place of Ser. Checking whether *Fitmunk* is able to place a conformation on a backbone and is able to solve the collisions with neighboring residues can therefore be used for the validation of main-chain placement, conformation and sequence assignment by ensuring that all side chains can be correctly packed. Selected cases for each type of validation that can be performed by *Fitmunk* are presented in Fig. 7 ▸.

## 4. Limitations and future developments   

The application of the algorithm presented in this work focuses on rebuilding the side chains on a fixed backbone that is already modeled and has assigned sequence. It is useful as a standalone tool to finalize and validate the model, but its use during the earlier stages of model building requires integration with other tools specializing in main-chain building and structure refinement. In our experience, the iterative use of *Fitmunk* and reciprocal refinement leads to improved electron-density maps and results in a better model fit and faster convergence, even with initial models of poor quality. *Fitmunk* can also be integrated with software for *de novo*/comparative modeling that will aid in rebuilding fragments without significant density; such integration would allow better modeling of ‘boundary regions’, in which the observed electron density is weak but still interpretable, and can strongly benefit from knowledge-based fitting.

In the present work, we have somewhat arbitrarily selected both the rotamer library that is used for deriving conformation frequencies and the force field for calculating interaction energies, and a thorough investigation of these two parameters is warranted. For example, using a backbone-dependent library such as that used in *Rosetta* (Shapovalov & Dunbrack, 2011 ▸) might be beneficial. We do not expect major differences in the final result, because the electron-density term dominates the energy function in most cases. However, the use of backbone-dependent frequencies in regions of poor electron density may increase the energy difference between different conformations and thereby reduce the time that is needed to find an optimal solution. Nevertheless, selection of the best rotamer library for frequency calculation and the best force field for *Fitmunk* requires additional, systematic studies.

## 5. Conclusions   

We have developed a new algorithm for amino-acid side-chain conformation modeling based on DEE theory, which is specifically tailored for applications in MX. The resulting implementation of the algorithm, in the form of the program *Fitmunk*, has been tested and evaluated both on newly collected data as well as on artificial test sets based on previously deposited structures. Evaluation of the program demonstrated that the DEE-based algorithm is both robust and effective, and can improve protein structures during the early and middle stages of model building and refinement. Specifically, *Fitmunk* was able to improve the fit of side chains, select better rotamers, resolve various cascading errors and significantly reduce the number of steric clashes, even in models already deposited in the PDB. Our evaluation of the program led us to the conclusion that some of the structures that have already been deposited can be improved by our program, especially those at medium and low resolutions. We have therefore investigated the possibility of using *Fitmunk* for model validation. We have demonstrated that *Fitmunk* can validate the selection of side-chain conformations and rotamers and help to answer the question whether a side chain is optimally fitted or could be fitted better. In conjunction with programs calculating local model to electron-density correlations, *Fitmunk* can be used to evaluate whether a side chain was unnecessarily removed from a model. The ability of *Fitmunk* to score very dense libraries also makes it a good tool to evaluate the probity of potentially problematic main-chain traces that lack side chains owing to low resolution or poor local electron density, and ultimately to support positioning the side chains to result in a properly packed fragment.

*Fitmunk* is available as a web service (at http://fitmunk.bitbucket.org/ or at http://kniahini.med.virginia.edu/fitmunk/server/) and can also be accessed in *HKL*-3000.

## Supplementary Material

Supplementary tables.. DOI: 10.1107/S2059798315024730/dz5393sup1.pdf

## Figures and Tables

**Table 1:** Comparison of the accuracy of *Fitmunk* and *MUMBO*

	PDB entry 2iim †		PDB entry 1thw		PDB entry 2hft		PDB entry 1dpx	
	χ_1_ < 20°	All χ_*i*_ < 20°	χ_1_ < 20°	All χ_*i*_ < 20°	χ_1_ < 20°	All χ_*i*_ < 20°	χ_1_ < 20°	All χ_*i*_ < 20°
*Fitmunk* ‡	98 (100)	84 (94)	92 (96)	81 (91)	95 (97)	84 (92)	97 (100)	86 (95)
*MUMBO*	92	76	85	67	82	70	89	72

† The structure reported in the *MUMBO* paper has not yet been published. This deposition has a matching protein and authors, but the resolutions reported in the PDB and the *MUMBO* paper differ (1.0 *versus* 1.3 Å).

‡ The values in parentheses show the percentage of correctly fitted residues classified after manual inspection. These included cases where the wrong conformation was selected in the original deposition, the orientations of Asn, Gln or His residues were corrected or a similarly fitting alternative conformation was selected.
