# Supplementary material for: Fitmunk: improving protein structures by accurate, automatic modeling of side-chain conformations
Source: Acta Crystallogr D Struct Biol. 2016 Jan 28;72(Pt 2):266–80. doi: 10.1107/S2059798315024730 (PMC4756610; doi:10.1107/S2059798315024730)
Supplement: Supplementary file 1 [file d-72-00266-sup1.pdf]

STRUCTURAL BIOLOGY

Volume 72 (2016)

Supporting information for article:

Fitmunk: improving protein structures by accurate, automatic modelling of side-chain conformations

Przemyslaw Jerzy Porebski, Marcin Cymborowski, Marta Pasenkiewicz-Gierula and Wladek Minor

Table S1 Comparison of R, R_{free}, *Molprobity* score and clashscore between structures from TS set refined and non-refined using *Fitmunk*.

Resolution bin	Median R factor difference	Median R_{free} factor difference	Median *Molprobity* score difference	Median clashscore difference
[0.9, 1.2]	-0.0008	-0.0001	-0.01	0.00
(1.2, 1.4]	-0.0010	0.0001	-0.07	-0.26
(1.4, 1.6]	-0.0011	0.0001	-0.05	-0.11
(1.6, 1.8]	-0.0013	0.0003	-0.14	-0.38
(1.8, 2]	-0.0014	-0.0004	-0.17	-0.69
(2, 2.2]	-0.0019	-0.0007	-0.17	-0.89
(2.2, 2.4]	-0.0025	-0.0009	-0.24	-1.40
(2.4, 2.6]	-0.0026	-0.0003	-0.28	-1.59
(2.6, 2.8]	-0.0023	-0.0012	-0.34	-2.85
(2.8, 3]	-0.0020	-0.0011	-0.40	-4.39
(3, 3.5]	-0.0008	-0.0007	-0.48	-5.64

Table S2 Comparison of accuracy, ED adjusted accuracy, percentage of sidechains with insignificant ED and percentage of residues improved by *Fitmunk*

Resolution bin	Median accuracy per structure [%]	Median ED adjusted accuracy per structure [%]	Median % of residues in a structure with insignificant density for sidechain	Median % of residues in a structure that were improved by *Fitmunk* in terms of density fit
[0.9, 1.2]	96.5	99.0	0.4	1.7
(1.2, 1.4]	96.0	99.4	0.6	2.6
(1.4, 1.6]	95.7	99.5	0.8	3.0
(1.6, 1.8]	93.8	99.4	1.0	4.5
(1.8, 2]	92.9	99.1	1.4	5.0
(2, 2.2]	90.1	98.6	2.2	6.5
(2.2, 2.4]	87.2	97.7	3.7	8.0
(2.4, 2.6]	83.5	97.3	6.0	10.1
(2.6, 2.8]	80.7	97.3	8.5	13.1
(2.8, 3]	75.2	96.6	16.1	14.4
(3, 3.5]	70.8	95.7	27.5	16.8
